# Differential Profiles of intensive care unit multidrug-resistant patients: Influence of prior antibiotic therapy on clinical features

**DOI:** 10.12669/pjms.41.3.10392

**Published:** 2025-03

**Authors:** Ramsha Ghazal Arshad, Kaleem Ullah Toori, Javeria Rahim

**Affiliations:** 1 Ramsha Ghazal Arshad, MBBS KRL Hospital, Islamabad, Pakistan; 2 Kaleem Ullah Toori, MBBS, MRCP, FRCP KRL Hospital, Islamabad, Pakistan; 3 Javeria Rahim, MBBS KRL Hospital, Islamabad, Pakistan

**Keywords:** Gram-negative infections, Intensive care units, Multidrug resistance

## Abstract

**Objectives:**

To study the characteristics and their influence on outcomes of ICU patients with multi drug resistant infections with and without prior antibiotic use before admission.

**Methods:**

This single center study included 365 patients admitted to Medical and Surgical ICUs of KRL Hospital, Islamabad, from January 2023 to January 2024, who acquired a multi drug resistant infection 48 hours post-admission to the ICU. This was an observational study and purposive sampling was done. Kolmogorov-Smirnov test was employed to test the normality of data. The chi-square test was used to observe the association between categorical variables. The Mann-Whitney U test was used for continuous variables. Multivariate analysis was employed to compare the effect of different parameters on mortality.

**Results:**

A total of 365 patients were included. The mean age was 62.2 ± 17.1, (<65 years = 54.2% and >65 years = 45.8%) with 185 (50.7%) males. Males, diabetics, those with chronic kidney disease, DCLD, CVA, hospitalization in last 6 months had a greater frequency of prior antibiotic exposure. Similarly, this group also showed increased frequency of thrombocytopenia and prolonged ICU stay than those with no previous antibiotic exposure. Longer duration of indwelling lines, hospital stay, ICU stay and Mechanical ventilation was associated with increased mortality.

**Conclusion:**

Previous antibiotic use is linked to longer ICU and hospital stays, extended use of indwelling lines, and increased duration of mechanical ventilation, all of which contribute to greater financial burdens. However, there was no significant difference in mortality between the antibiotic and non-antibiotic groups. Further studies conducted on a larger scale across multiple ICUs could provide deeper insights into this relationship.

## INTRODUCTION

Antimicrobial resistance (AMR) has a profound impact on clinical outcomes for patients in intensive care units (ICUs).^1^ Despite the well-established benefits of antimicrobial therapy, the associated risks are often overlooked. The World Health Organization (WHO) recognizes the global proliferation of antibiotic resistance as a health crisis that demands urgent intervention.^2^ in 2019, Pakistan ranked 176th out of 204 countries in terms of AMR-related mortality per 100,000 people, making AMR the third leading cause of death in 2019.^3^

The rise in resistance complicates the management of nosocomial infections, which contribute significantly to ICU patient morbidity and mortality.^4^ Excessive antibiotic use, both in and out of ICUs, extends hospital stays and increases costs.^5^,^6^ Limited awareness of AMR among healthcare providers and the public leads to improper antibiotic use, accelerating resistance.^7^ Additionally, pre-existing comorbidities in patients increase the likelihood of recurrent infections, driving further antibiotic overuse.^8^

Regarding the pathogens responsible for ICU infections, Gram-negative bacteria are commonly isolated, with Klebsiella species, *Escherichia coli*, *Pseudomonas aeruginosa*, and *Acinetobacter baumannii* being most prevalent.^9^,^10^ Local studies confirm similar findings.^11^-^13^ Gram-positive organisms follow closely, with *Staphylococcus aureus* being the most frequently detected, including 7.4% methicillin-sensitive, 4.7% methicillin-resistant, linezolid- or vancomycin-resistant, and 4.6% methicillin-resistant *S. aureus*.^14^

Healthcare-associated infections are two to three times more common in developing countries like Pakistan, with ICU-acquired infections being more prevalent than in other hospital departments.^13^,^15^-^17^ This is largely due to the severity of patient conditions and prolonged ICU stays. Contributing factors include immunosuppression, multiple comorbidities, frequent antibiotic use, and reliance on invasive devices like central venous catheters, urinary catheters, and mechanical ventilation, all of which increase the risk of multidrug-resistant infections.^18^

There is scarcity of data in the local population, regarding risk factors for MDR ICU infections and those determining adverse outcomes. This study aimed to identify patient characteristics and their influence on outcomes in ICU patients with multi-drug resistant infections, with and without prior antibiotic use before admission

## METHODS

This study was a single center, observational study conducted at KRL General Hospital, Islamabad, Pakistan from January 2023 to January 2024.

### Ethical Approval:

The Institutional Review Board (IRB) approval was taken on 15th December 2022 (Ref ERC: KRL-HI-PUB0ERC/May22/10, Dated: December 15, 2022) and consent was taken from individuals or their legal guardians before commencing.

### Inclusion criteria:

• All patients acquiring a multi-drug-resistant organism (MDRO) 48 hours after admission to Medical or Surgical ICU, over 14 years of age.

### Exclusion criteria:

• Those patients admitted in wards or out-patients and whose cultures do not show growth of MDRO.

### Outcome

Outcome was defined as length of ICU stay and mortality.

### Operational definition:

MDRO: microorganism, predominantly bacteria that cause infections that are non-susceptible to at least one agent in three or more antimicrobial categories.^17^

The sample size was calculated to be 365, by the Raosoft calculator by using the reference proportion of 41%.^18^

The data was collected at admission and included demographic characteristics such as age, gender, and existing medical conditions. Comprehensive baseline assessments were conducted, including complete blood counts, liver and renal function tests (RFTs), and electrolyte levels, in addition to CRP measurements and cultures from blood, urine, sputum or bronchial washings, pus from bed sores or infected wounds after 48 hours of ICU admission. Only MDR patients were included and comparison was done based on prior antibiotic use in the last six months.

### Statistical analysis:

Total 365 patients were enrolled, data was taken over one year, and SPSS ver. 23 was used to analyze the variables. Frequencies were computed for qualitative variables such as gender and, the Kolmogorov-Smirnov test was employed to assess the normal distribution of data which yielded a value of 0.324 with a p-value of <0.01. The chi-square test assessed the proportions between categorical variables and Mann-Whitney U test to compare continuous data. A *p*-value of <0.05 with a confidence interval of 95% was taken as significant. Univariate analysis was used to model outcomes with clinical characteristics and values <0.05 were taken for multivariate logistic analysis to address the confounders.

## RESULTS

Out of the 365 patients, 197(54%) had a history of prior antibiotic use in the last six months. The mean age was 62.2 ± 17.1 years (<65 years = 54.2% and >65 years = 45.8%) with185 (50.7%) males. The rest of the baseline characteristics are listed in Table-I. The two groups (patients with or without prior antibiotics use) had no difference in the need for mechanical ventilation. The analysis also showed that elderly, males, diabetics, those with CKD, DCLD, CVA, and previous hospitalization had more incidence of antibiotics use . The same cohort also showed an increased frequency of thrombocytopenia and deranged renal functions. The Mann-Whitney U test showed a statistically significant difference between the total duration of ICU stay and previous antibiotic use (*p*=0.016), and slightly increased number of mortality in antibiotic group (55% vs 45%) however, this difference was not statistically significant (*p*=0.75).

**Table-I:** Baseline characteristics of the study population.

VARIABLE	Overall Group	No prior Antibiotic use	Prior Antibiotic use	Pearson’s Chi-Square	p-value
***Age***	62.2+17.1	168(46%)	197(54%)	8.5	0.004**
<65	198	105(62.5%)	93(47.2%)		
>65	167	63(37.5%)	104(52.8%)		
***Gender***		168(46%)	197(54%)	4.5	0.03*
Males	185	75(44.6%)	110(55.8%)		
Females	180	93(55.4%)	87(44.2%)		
***Comorbidities***					
***a) Pre-admission variables***					
Hypertension	254(69%)	109(64.9%)	145(73.6%)	3.26	0.08
Diabetes	250(68.5%)	106(63.1%)	144(73.1%)	4.2	0.04*
IHD	131(35.8%)	55(32.7%)	76(38.6%)	1.34	0.27
CKD	107(29.3%)	38(22.6%)	69(35%)	6.73	0.01*
DCLD	16(4.4%)	3(1.8%)	13(6.6%)	5.01	0.03*
CVA	49(13.4%)	12(7.1%)	37(18.8%)	10.5	0.001**
Malignancy	15(4.1%)	5(3%)	10(5.1%)	1.01	0.43
Previous hospital admission	131(35.8%)	15(8.9%)	116(58.9%)	98.3	0.000**
***b) Post-admission variables***					
Mechanical Ventilation	149(40%)	73(49%)	76(51%)	0.89	0.201
Duration of indwelling lines	15.7+12.4	168(46%)	197(54%)		<0.001**
Inotropic support	159(43.5%)	66(39.3%)	93(47.2%)	2.31	0.14
Steroid use	77(21%)	29(17.3%)	48(24.4%)	2.75	0.122
Hemoglobin	10.7+2.1	121(72%)	154(78.2%)	1.84	0.18
TLC >10,000	15.2+5.7	152(90.5%)	175(89%)	0.26	0.73
Platelets	233+82	19(11.3%)	38(19.3%)	4.3	0.04*
Liver dysfunction		7(4.2%)	9(4.6%)	0.03	1.0
Renal dysfunction		102(60.7%)	144(73.1%)	6.33	0.014*
Coagulation dysfunction		13(6.6%)	13(6.6%)	0.17	0.41
Duration of ICU stay	12.2+8.7	10.8 +5.9	13.4+10.3		0.016*
Duration of hospital stay	14.9+10.1	168(46%)	197(54%)		0.053
Mortality	139(38%)	62(45%)	77(55%)	0.18	0.75

* Significant at <0.05

** Significant at <0.01.

Univariate logistic regression analysis of all variables was done and variables with a *p*-value <0.05 were selected for multivariate logistic regression analysis in Table-II. The final model revealed that deranged coagulation, duration of mechanical ventilation, indwelling lines, and prolonged ICU and hospital stays were associated with increased mortality odds in MDR ICU patients, with ICU stay being 28 times more predictive than the other factors Table-III.

**Table-II:** Univariate binary logistic regression.

Variables	OR (95% CI)	*p*-value
Age	0.23(0.12-0.43)	0.00*
Gender	0.84(0.47-1.50)	0.55
Hypertension	2.08(1.04-4.17)	0.04*
Diabetes	1.51(0.78-2.94)	0.22
IHD	2.52(1.39-4.56)	0.002
CKD	2.79(1.53-5.12)	0.001
DCLD	1.36(0.44-4.22)	0.59
CVA	3.73(1.76-7.89)	0.001
Malignancy	2.45(0.67-8.98)	0.18
Overall premorbids	2.12(1.56-2.87)	0.000
Previous hospital admission	2.67(1.44-4.94)	0.002
Mechanical Ventilation	15.1(7.43-30.8)	0.000
Duration of indwelling lines	5.09(3.27-7.94)	0.000
Inotropic support	0.82(0.46-1.45)	0.49
Steroid use	1.81(0.94-3.50)	0.07
Hemoglobin	0.39(0.18-0.85)	0.02
TLC>10,000	1.34(0.55-3.28)	0.52
Platelets	0.75(0.37-1.53)	0.43
Liver dysfunction	1.29(0.35-5.35)	0.72
Renal dysfunction	0.41(0.20-0.82)	0.012
Coagulation dysfunction	0.05(0.006-0.35)	0.003
Duration of ICU stay	1.06(1.02-1.09)	0.001
Duration of MV	1.14(.09-1.20)	0.000
Duration of Hospital stay	1.03(1.003-1.060)	0.03

* Significant at <0.05.

**Table-III:** Multivariate logistic regression.

Variables	Regression coefficient	OR(95%CI)	*p*-value
Age	-1.633	0.19(0.03-1.39)	0.10
Hypertension	-.065	0.94(0.18-4.8)	0.94
IHD	-.194	0.82(0.15-4.5)	0.82
CKD	0.711	2.03(0.50-8.2)	0.32
CVA	1.282	3.60(0.65-19.9)	0.14
Total premorbids	-.066	0.94(0.38-2.72)	0.90
Mechanical Ventilation	-1.14	0.32(0.02-6.89)	0.47
Hemoglobin	1.073	2.92(0.29-26.5)	0.36
Renal dysfunction	-0.499	0.61(0.09-4.21)	0.61
Duration of Indwelling lines	-1.23	0.29(0.10-0.83)	0.02
Coagulation dysfunction	-10.21	0.00(0.00-0.15)	0.001
Duration of hospital stay	-2.09	0.12(0.03-0.46)	0.002
Duration of Mechanical ventilation	0.62	1.85(1.25-2.74)	0.002
Duration of ICU stay	3.33	28(4.81-162)	0.000

## DISCUSSION

Various factors have been identified for the acquisition of multi-drug resistant infections over the past decade. Notable causes include the increasing empirical use of antibiotics, recurrent hospitalizations, previous immunocompromised status, and any invasive procedures.^19^-^21^ In this study, we evaluated both the pre-and post-admission variables in patients with previous antibiotic use, as independent risk factors for MDR. Our findings indicate that age, gender, diabetes, kidney and liver diseases, along with previous hospitalizations were associated with more antibiotic use.

In our study, we observed that the use of antibiotics was associated with an increase in the length of ICU stay (*p*=0.005), as reported in the previous literature.^22^ Similarly, patients with prior comorbidities like diabetes, CVA, chronic kidney disease, or liver disorders had more frequent antibiotic use.^23^,^24^ Hypertension was the most frequent comorbid illness seen in our cohort (69%), while malignancy had the lowest frequency (4.1%).This difference could have been the reason that there was no significant use of antibiotic use in this group, as contrary to the present literature.^25^

Our study also showed that males had a higher percentage of antibiotics use (Male=56% vs Female=44%) and this difference was statistically significant (*p*=0.03). This difference could be attributed to variable health-seeking behaviours and access to healthcare resources in between the two genders.^26^ Further research is necessary to determine the cause of the gender differences in the prevalence of MDR infections and frequent antibiotic use. Additionally, we also noted more antibiotic use prior to hospitalization in patients with >65 years of age (>65=53% vs ≤65=47%) and this difference between the two age groups was statistically significant (*p*=0.004). This is likely due to prevalence of multiple comorbidities and higher disease severity in this age group.^27^

The biochemical parameters of our patients indicated that only thrombocytopenia was more common in those with a history of antibiotic use (19.3%). This could be attributed to the side effects of antibiotics, anticoagulation therapy, or sepsis itself. A retrospective study in China by Zhang et al showed that linezolid use was more associated with thrombocytopenia than glycopeptides.^28^ Similarly, Ostadi et al described multiple mechanisms that can contribute towards thrombocytopenia in an ICU setting.^29^ Although our study did not show a significant association of low platelet counts with mortality, further studies might be needed to assess the correlation of this parameter with disease progression or poor patient outcomes.

In this study, out of 365 patients, 159 required inotropic support and among these, 58% of patients had a history of antibiotic use. Similarly, only 77 patients required steroids amongst which 62% of patients belonged to the previous antibiotic use group. This indicated a positive trend that was following previous studies.^30^ These results however were not statistically significant (*p* >0.05). Our study found no significant difference between previous antibiotic use and patient mortality, which contradicts findings from earlier literature.^31^ This discrepancy may be due to the in-hospital prescription of empiric broad-spectrum antibiotics, tailored to the hospital’s antibiogram or due to a lesser number of patients in both cohorts.

**Table:** 

PERFORMA USED	
ID:	
Age:	
Gender:	
Comorbids:	
Diagnosis:	
Date of admission:	
Antibiotic in last 06 months and duration (if any):	
Antibiotics used in hospital stay in current admission:	
Organism grown (if any) and culture:	
Previous hospital admissions in last 06 months:	
Mechanical Ventilation:	
Cleaning of vents:	
Outcome:	
Duration of ICU stay:	
Mortality:	
Lab parameters:	
Hemoglobin:	
TLC:	
Platelets:	
CRP:	
LFTs:	
RFTs:	
Electrolytes:	
PT/APTT/INR:	
Urine R/E:	
CXR:	

However, multivariate analysis revealed that coagulation dysfunction, duration of indwelling lines, duration of mechanical ventilation, length of ICU and hospital stays were independent risk factors for poor outcome in patients with multidrug-resistant (MDR) infections.^31^,^32^ The strength of our study is the use of simple patient clinical and biochemical parameters as tools to predict poor outcome in ICU MDR patients, exposed to previous antibiotics.

### Limitations:

Despite these encouraging results, it is important to acknowledge the limitations of our study. This was a single-center, cross-sectional observational study that interfered with the generalizability of the results and purposive sampling could potentially introduce bias. The variables were taken only at one point in time, that was, after 48 hours of hospital admission. Follow-up parameters and response to treatment measures done during ICU stay were not assessed at discharge or death. Only MDR patients were taken from a single ICU and the findings may not be applicable to other settings. However, we assumed that our sample was representative of our local population and that further studies in ICUs at regional and national levels can help gain more understanding of these factors with MDR infections.

## CONCLUSION

This study highlights the association between antibiotic use, patient characteristics, and clinical outcomes. Previous antibiotic use is linked to longer durations of mechanical ventilation, extended use of indwelling lines, and increased ICU and hospital stays, all of which place significant strain on hospital resources. Implementing effective antibiotic stewardship and local ICU policies can help manage infections more effectively and reduce the associated economic burden. Although no association with mortality was found between the two groups, further large-scale studies across various ICUs nationwide could provide a clearer understanding of the relationship between antibiotic use and mortality.

### Author`s Contribution:

**RGA:** Acquisition of data, data analysis and manuscript writing.

**KUT:** Conception and design, critical revision of the data, responsible for accuracy of the data, final approval before publication.

**JR:** Acquisition of data, critical review and manuscript writing.
